# Cell-Based and Pharmacologic Hormone Therapy Maintain Diastolic Function After Ovariectomy in Hypertensive Rats

**DOI:** 10.1093/geroni/igaa057.429

**Published:** 2020-12-16

**Authors:** Leanne Groban, Xuming Sun, Sittadjody Sivanandane, Hunter Hodge, Hao Wang, Marina Lin, Emmanuel Opara

**Affiliations:** Wake Forest School of Medicine, Winston-Salem, North Carolina, United States

## Abstract

The role for hormone therapy in the maintenance of diastolic function upon ovarian senescence has not been clinically tested due to concerns for off-target health risks. We developed a cell-based hormone therapy (cHT) approach that recapitulates native cell–cell interactions between ovarian granulosa and theca cells in a 3D bioengineered construct to mimic the dynamic release of sex hormones. Our first report in ovariectomized (OVX) rats shows that cHT ameliorates various adverse somatic effects of hormone deficiency (e.g. bone loss). To extend these findings to cardiac health, we sought to determine the efficacy of cHT in preserving diastolic function in OVX-spontaneously hypertensive rats (SHR). 14 SHRs underwent bilateral OVX while 5 SHRs received sham surgery at 12 weeks of age. Following an 8-week washout, OVX rats were randomized to cHT or pharmacologic hormone therapy (pHT: E2 (10 mcg/kg/day) and P4 (2 mg/kg/day, s.c.) for 10 weeks and compared to OVX-vehicle and Sham. While uterine atrophy by OVX was minimized by cHT and pHT, hormone levels across OVX groups were not overtly different. Systolic blood pressure increased progressively over time (P<0.01), without a treatment effect. Even so, cHT and pHT prevented OVX-related reductions in myocardial relaxation and increases in Doppler-derived filling (P<0.05); paralleling the diastolic profile of Sham. Alongside superior diastolic function, 25% increases in cardiac interferon regulatory factor-4 (Irf-4) gene expression levels occurred in both hormone-treated OVX groups and Sham when compared to OVX-vehicle, suggesting a link between sex hormones and local immune modulation in the regulation of female cardiac health.

